# Supplementary material for: Amelioration of Nicotine-Induced Conditioned Place Preference Behaviors in Mice by an FABP3 Inhibitor
Source: Int J Mol Sci. 2023 Apr 2;24(7):6644. doi: 10.3390/ijms24076644 (PMC10095245; doi:10.3390/ijms24076644)
Supplement: Supplementary file 1 [file ijms-24-06644-s001.zip › ijms-2284855-supplementary.pdf]

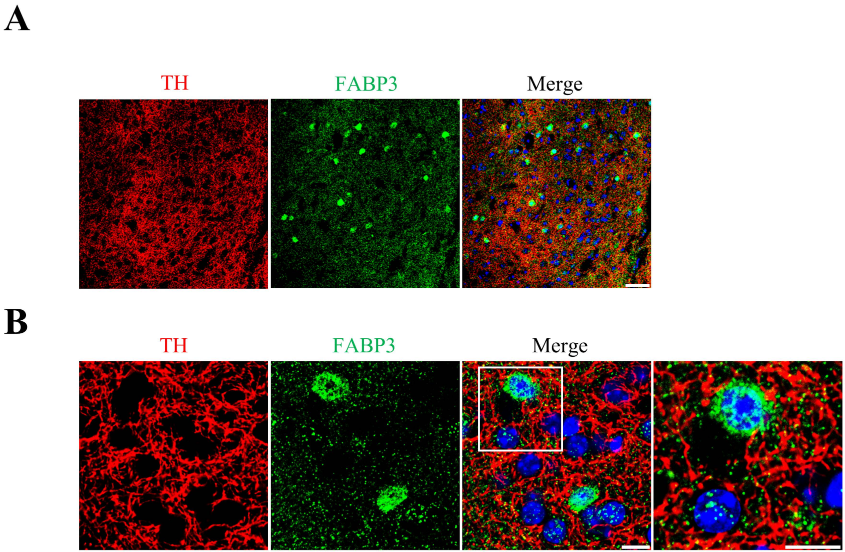

Supplementary Figure. S1. The double staining of FABP3 and TH in the NAc. **A** Representative images of TH (red) and FABP3 (green) double staining in mouse NAc (n = 5 per group). *Scale bars*, 50 μm. **B** Representative magnified images of TH and FABP3, respectively. TH-positive terminals but not TH-positive cells were clearly observed in the NAc. DAPI-stained nuclei are shown in blue color. *Scale bars*, 10 μm.
